# Diverse Exopolysaccharide Producing Bacteria Isolated from Milled Sugarcane: Implications for Cane Spoilage and Sucrose Yield

**DOI:** 10.1371/journal.pone.0145487

**Published:** 2015-12-28

**Authors:** Stanton Hector, Kyle Willard, Rolene Bauer, Inonge Mulako, Etienne Slabbert, Jens Kossmann, Gavin M George

**Affiliations:** 1 Institute for Plant Biotechnology, Department of Genetics, Stellenbosch University, Private Bag X1, Matieland, 7602, South Africa; 2 LaunchLab, Stellenbosch University, Private Bag X1, Matieland, 7602, South Africa; 3 Department of Microbiology, Stellenbosch University, Private Bag X1, Matieland, 7602, South Africa; 4 Central Analytical Facility, Stellenbosch University, Private Bag X1, Matieland, 7602, South Africa; ContraFect Corporation, UNITED STATES

## Abstract

Bacterial deterioration of sugarcane during harvesting and processing is correlated with significant loss of sucrose yield and the accumulation of bacterial polysaccharides. Dextran, a homoglucan produced by *Leuconostoc mesenteroides*, has been cited as the primary polysaccharide associated with sugarcane deterioration. A culture-based approach was used to isolate extracellular polysaccharide (EPS) producing bacterial strains from milled sugarcane stalks. Ribosomal RNA sequencing analysis grouped 25 isolates into 4 genera. This study identified 2 bacterial genera not previously associated with EPS production or sucrose degradation. All isolates produced polysaccharide when grown in the presence of sucrose. Monosaccharide analysis of purified polymers by Gas Chromatography revealed 17 EPSs consisting solely of glucose (homoglucans), while the remainder contained traces of mannose or fructose. Dextranase treatment of polysaccharides yielded full digestion profiles for only 11 extracts. Incomplete hydrolysis profiles of the remaining polysaccharides suggest the release of longer oligosaccharides which may interfere with sucrose crystal formation.

## Introduction

Sugarcane is the most important crop for the production of sucrose and, increasingly, bioethanol [1]. The estimated gross annual production of sucrose from sugarcane is valued at $76 billion (Food and Agricultural Organization of the United Nations; http://faostat.fao.org). During production, sucrose and contaminating sugars are precipitated from juice released from crushed sugarcane stalks [2–4]. Cut sugarcane is stored at ambient temperature for an average of 3–5 days before processing [4, 5]. This ‘cut-to-crush delay’ allows for losses as high as 20–30% of extractable sucrose with a concomitant accumulation of bacterial exopolysaccharides (EPS) [4, 6–8]. Cut sugarcane deterioration is influenced by several abiotic and biotic factors and is exacerbated by high ambient temperatures and rainfall [5, 7, 9]. Sucrose degradation is mainly due to bacterial metabolism and chemical inversion [4]. Indeed, Eggleston [7] showed that 95% of sucrose loss can be attributed to bacterial spoilage.

A number of microorganisms secrete enzymes which utilise sucrose as substrate for synthesis of oligo- and polysaccharides, releasing monosaccharides as a carbon source. The impact of EPS on the production of sugar is an industrial concern due to raised viscosity of the massecuite during processing, which inhibits evaporation and crystal formation [2, 9–15].

Dextran is synthesized by an extracellular dextransucrase enzyme, using sucrose as the sole substrate. Bacterial dextran consists of α-(1,6) linked glucose polymers with α-(1,3) or occasionally α-(1,4) or α-(1,2) branched linkages with molecular weights up to several million Daltons [2, 16]. Dextran has been shown to be the most problematic and abundant EPS produced during sugarcane deterioration [4, 17, 18]. Accumulation of the polysaccharide in sugarcane juice during processing can be controlled through good management practises and the use of the enzyme dextranase [4, 12]. Complete hydrolysis of dextran by dextranase yields oligomers of between 2–10 glucose units and reduces the viscosity of massecuite [17].

Dextran, produced by *Leuconostoc mesenteroides*, has been cited as the primary contaminating EPS accumulating during sugarcane deterioration [4, 7, 12, 17, 18]. Prevention of microbial growth during sugarcane milling is necessary in order to maintain high yields. Several management and remediation strategies have been reviewed where the importance of optimal cutting practises and the minimization of time between cutting and processing were emphasised to reduce bacterial spoilage [4]. Other EPS producing microorganisms such as *Penicillium* sp., *Streptococcus* spp., *Lactobacillus* spp. [19], *Xanthomonas albilineans* [20] and *Acetobacter diazotrophicus* [21] were shown to be present at cut ends and damaged sites of the cane after harvesting. Efforts to reduce the problem of bacterial polysaccharide accumulation in harvested sugarcane are hindered by the current lack of knowledge regarding the diversity of microorganisms involved in this process. Furthermore, the presence of bacterial species producing EPS other than dextran is not addressed in current strategies for treatment of deteriorated sugarcane.

This study investigates the culturable EPS-producing bacterial diversity associated with sugarcane during processing. Monosaccharide compositions of polysaccharides, the relative concentration of EPS produced on different sugars, as well as sensitivity to dextranase, are reported.

## Materials and Methods

### Preparation of milled sugarcane

Sugarcane stalks were cut below ground level, the tops removed, and stalks stacked outside in bundles for 3 days at the South African Sugarcane Research Institute (SASRI) laboratory in Durban, South Africa. Average temperature and humidity values (day/night) during storage were 26°C/19°C and 94%/59%, respectively. The cut-ends of over 30 stalks were randomly selected from a pool of several hundred and were blended with double the volume of water and filtered through a mesh funnel. The milled filtrate was cooled to 20°C and passed through filter paper containing 3 g of celite.

### Selection of EPS producing isolates and relative polysaccharide production

The milled filtrate was diluted and plated onto De Man, Rogosa and Sharpe (MRS) (Merck, Darmstadt, Germany) and Luria Bertani (LB) (Merck, Darmstadt, Germany), both supplemented with 2% sucrose, and incubated at 30°C for 48 hrs to allow for sufficient polysaccharide production. Isolates first recovered from MRS or LB medium were designated as SM or SL respectively. EPS production was confirmed visually or through the string test [22]. The formation of a string (>5 mm) upon lifting of the loop was considered positive.

Single colonies from each isolate were streaked onto SDM [23] supplemented with 2% (m/v) sucrose, glucose or fructose. EPS production was assessed after incubation for 16 h at 22°C (Table 1, S1 Fig).

**Table 1:** EPS producing isolates nearest type strains, GenBank accession, string test results, monosaccharide composition, dextranase susceptibility and relative polysaccharide abundance are shown.

Family	Nearest type strain	GenBank accession	Isolate	String test positive^a^	Digested by Dextranase (*Chaetomium erraticum*)^b^	EPS monosaccharide composition from isolates grown on sucrose				Relative EPS abundance when grown on different sugars		
						Glc	Gal	Fru	Man	Suc	Glc	Fru
Leuconostocaceae	*Lueconostoc lactis*	KU060301	SM33	-	++	✓				✓		
	*Lueconostoc citreum*	KU060286	SL8	-	+	✓			✓	✓		
	*Lueconostoc citreum*	KU060287	SL27	-	+	✓				✓		
	*Lueconostoc citreum*	KU060303	SM19	-	+	✓			✓	✓		
	*Lueconostoc citreum*	KU060299	SM40	-	+	✓			✓	✓		
	*Lueconostoc citreum*	KU060293	SM20	-	+	✓				✓		
	*Lueconostoc citreum*	KU060297	SL26	-	++	✓				✓		
	*Lueconostoc citreum*	KU060307	SM5	-	+	✓				✓		
	*Leuconostoc sp*.	KU060295	SM36	-	++	✓				✓		
	Uncultured Leuconostoc	KU060285	SL29	-	+	✓				✓		
	*Lueconostoc citreum*	KU060289	SL10	-	++	✓				✓		
	*Lueconostoc citreum*	KU060288	SM7	-	+	✓			✓	✓		
	*Lueconostoc citreum*	KU060298	SL19	-	+	✓		✓		✓		
	*Lueconostoc citreum*	KU060284	SL25	-	+	✓			✓	✓		
	*Lueconostoc citreum*	KU060290	SM31	-	++	✓				✓		
	*Lueconostoc citreum*	KU060302	SM16	-	+	✓			✓	✓		
	Uncultured Weissella	KU060304	SL3	-	++	✓				✓		
Leuconostocaceae	*Weissella confusa*	KU060296	SM32	-	++	✓				✓		
	*Weissella cibaria*	KU060292	SL2	-	++	✓				✓		
	*Weissella cibaria*	KU060305	SL13	-	++	✓				✓		
	*Weissella confusa*	KU060300	SM10	-	++	✓				✓		
Lactobacillaceae	*Lactobacillus satsumensis*	KU060306	SM34	-	+	✓				✓		
	*Lactobacillus satsumensis*	KU060283	SM38	-	+	✓				✓	✓	
Enterobacteriaceae	*Salmonella bongori*	KU060294	SL18	+	+	✓			✓	✓	✓	✓
	*Salmonella bongori*	KU060291	SL9	+	++	✓				✓	✓	✓

^a^ Isolates selected for string test (+) or EPS production (-)

^b^ Purified EPS digested overnight to evaluate the sensitivity to dextranase (- indicates no digestion; + indicates partial digestion; ++ indicates full digestion)

### 16S rRNA gene sequence analysis

Genomic DNA was extracted according to Babalola *et al*. [24] and used as a template for 16S rRNA gene amplification. Amplicons were generated using KAPA HiFi™ (KAPA Biosystems, Woburn, USA) and universal 16s rRNA gene primers E9F (5’-GAGTTTGATCCTGGCTCAG-3’) and 1512R (5’- ACGGCTACCTTGTTACGACTT-3’) [25, 26] with an annealing temperature of 55°C for 25 cycles. Amplicons were cloned by ligation into pJET 1.2™ (Fermentas, Burlington, Ontario, Canada) and sequenced using BigDye terminator V3.1. Post sequencing clean-up was done using Centri-sep columns prior to analysis on a Life Technologies 3730xl sequencer. Phylogenetic analysis of the 16S rDNA sequence of strains were performed by using Mega 6.0 software package [27]. The consensus sequence and the sequences of strains belonging to *Leuconostoc*, *Lactobacillus*, *Weissella* and *Salmonella*, retrieved from the NCBI GenBank database, were aligned. The phylogeny was inferred using the Neighbor-Joining method [27]. The percentage of replicate trees in which the associated taxa clustered together in the bootstrap test (1000 replicates) are shown [28]. The evolutionary distances were computed using the Maximum Composite Likelihood method [29] and are in the units of the number of base substitutions per site. All positions containing gaps and missing data were eliminated. There were a total of 1321 positions in the final dataset.

### Purification of the EPS

EPS was isolated from cultures grown in semi-defined medium (SDM) supplemented with 5% sucrose [23] at 22°C. EPS purification was performed according to Bauer *et al*. [23] with slight modification. Isolated EPS was resuspended in 5 ml MilliQ (MQ) water (Millipore, Bilerica, MA, USA) and dialysed overnight in SnakeSkin® dialysis tubing (MWCO 3500 kDa) (ThermoScientific, Rockford, IL, USA) against 20 l of MQ water. EPS samples were freeze-dried on a BenchtopK (VirTis, Warminster, PA, USA) for 24h and stored at -20°C.

### Gas chromatography-based analysis of the EPS monosaccharide composition

Purified polysaccharide (2 mg) was hydrolysed and derivatized according to Roessner *et al*. [30]. Gas Chromatography (GC) was used to determine monosaccharides present in the EPS hydrolysate. Glucose, galactose, mannose and fructose were used as standards. A combination of GC-flame ionization detector (GC-FID) and GC-mass spectrometry (GC-MS) was used. A Hewlett Packard 4550 GC-FID system fitted with an auto sampler and Rtx^®^-5MS (30 m by 0.25 mm by 0.25 μm film thickness) column was used. The GC operating conditions were as follows: injection port temperature, 280°C; detector temperature, 250°C; initial oven temperature, 120°C; hold for 0 min; first ramp 10°C/min to 160°C; hold for 0 min; second ramp 1.5°C/min to 220°C; hold for 0 min; third ramp 20°C/min to 280°C; hold for 3 min; flow rate, helium column, ca. 1 ml/min; injection mode split less.

GC-FID results were confirmed by GC-MS analysis of the EPS produced by an isolate from each genus. Samples were analysed with an Aligent Technologies (Agilent Technologies, Santa Clara, CA) 6890N Network GC system coupled to a 5975 inert Mass Selective Detector. Electron impact ionization was performed at 70 eV. GC-MS conditions were the same as Bauer *et al*. [23].

### Dextranase treatment

Purified polysaccharide (10 mg) was resuspended in 1 ml of 50 mM sodium phosphate buffer (pH 6) containing ~5 KDU *C*. *erraticum* dextranase D0443 (Sigma-Aldrich, St. Louis, MO, USA) and incubated at 55°C for 16 h. The dextranase-treated polysaccharide was concentrated using a GeneVac EZ2 bench top evaporator to a final concentration of 0.1 mg/μl. The effect of the dextranase treatment was visualised using Thin Layer Chromatography (TLC). The assay was optimized on Dextran T500 (GE Healthcare Bio-Sciences AB, Uppsala, Sweden).

A total of 10 μg enzymatically-hydrolysed polysaccharide samples were spotted adjacent to non-hydrolysed polysaccharide on a silica gel 60 (F_254_) TLC plate (Merck, Darmstadt, Germany). Glucose, maltose, maltotriose and dextrimaltose were used as standards. The mobile phase consisted of 2:5:1.5 (by volume) acetic acid:1-propanol:water [31]. Plates were sprayed with sulphuric acid (5%) in ethanol and developed at 100°C for 10 min.

### Nucleotide sequence accession number

All the 16S rRNA gene sequences obtained have been submitted to GenBank database under accessions KU060283-KU060307 (See Table 1).

## Results and Discussion

**Fig 1:**
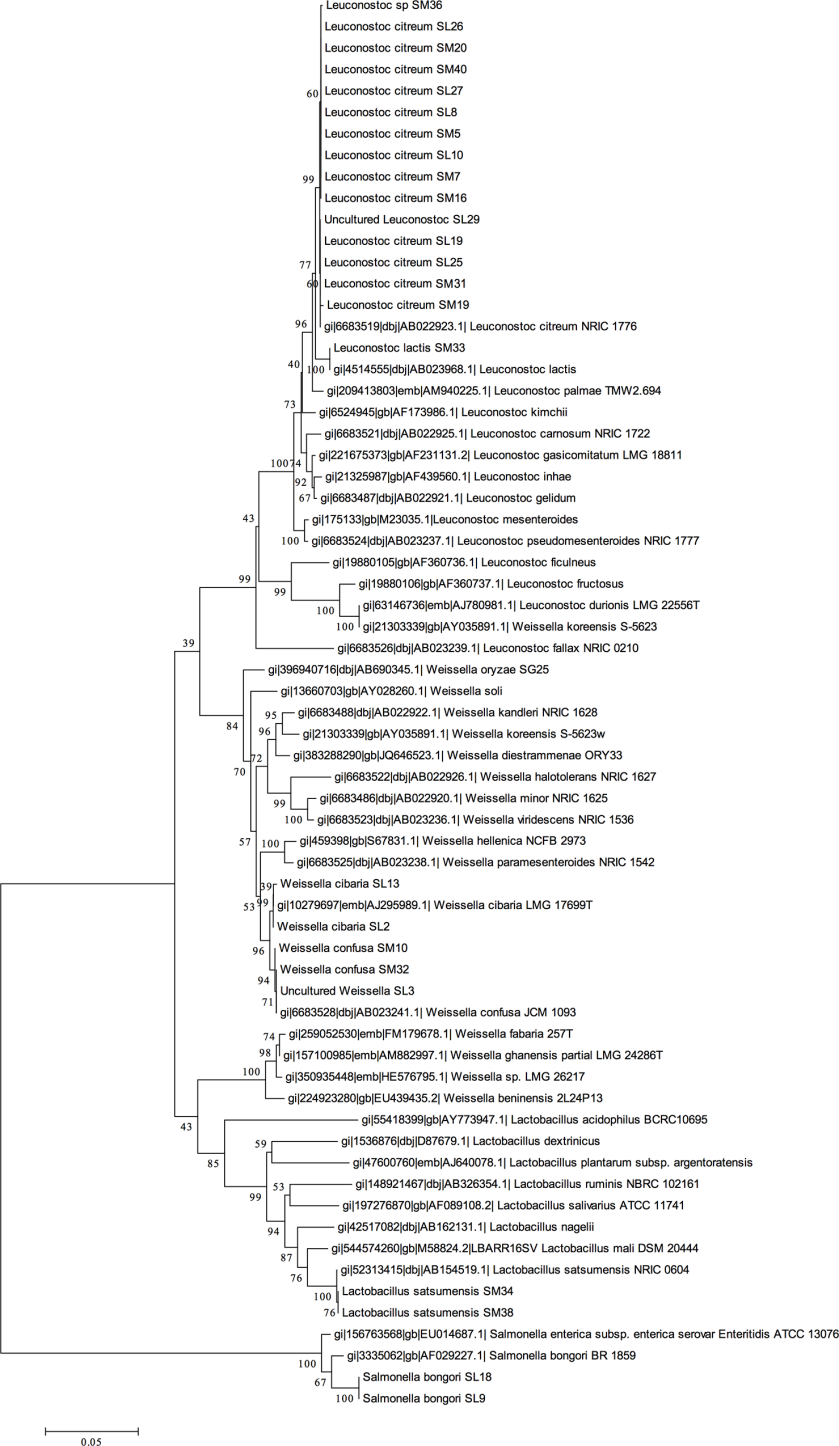
Population structure of polymer producing bacterial species. Condensed neighbour-joining phylogenetic tree of isolates identified in milled sugarcane. Homologies of 1321 bp sequences in the 16S rDNA of *Leuconostoc*, *Weissella*, *Lactobacillus* and *Salmonella* type strains are displayed along with sugarcane associated isolates.

The presence of EPS in sugar mills has mainly been associated with bacterial degradation of sucrose. This industrially and financially detrimental process has been attributed mainly to *Leuconostoc* and *Weissella* spp., which have been shown to be involved in sugar degradation and subsequent EPS formation [4, 32]. In order to probe the depth of microorganism diversity that may impact sucrose processing, we employed a culture-based screen for bacterial colonies which showed mucoid secretion when supplied only with sucrose. The bacterial strains were isolated from milled sugarcane obtained from the South African Sugarcane Research Institute (SASRI). Based on morphological characteristics, initial selection pointed to a wide variety of isolates, however, 16S rRNA gene sequencing and phylogenetic analysis revealed the distribution of bacteria were limited to only 4 genera (Fig 1). While the population was dominated by *Leuconostoc* and *Weissella* spp., isolates with highest sequence homology to *Lactobacillus satsumensis* (99%) and *Salmonella bongori* (99%), organisms novel to sugarcane processing environs, were present.

**Fig 2:**
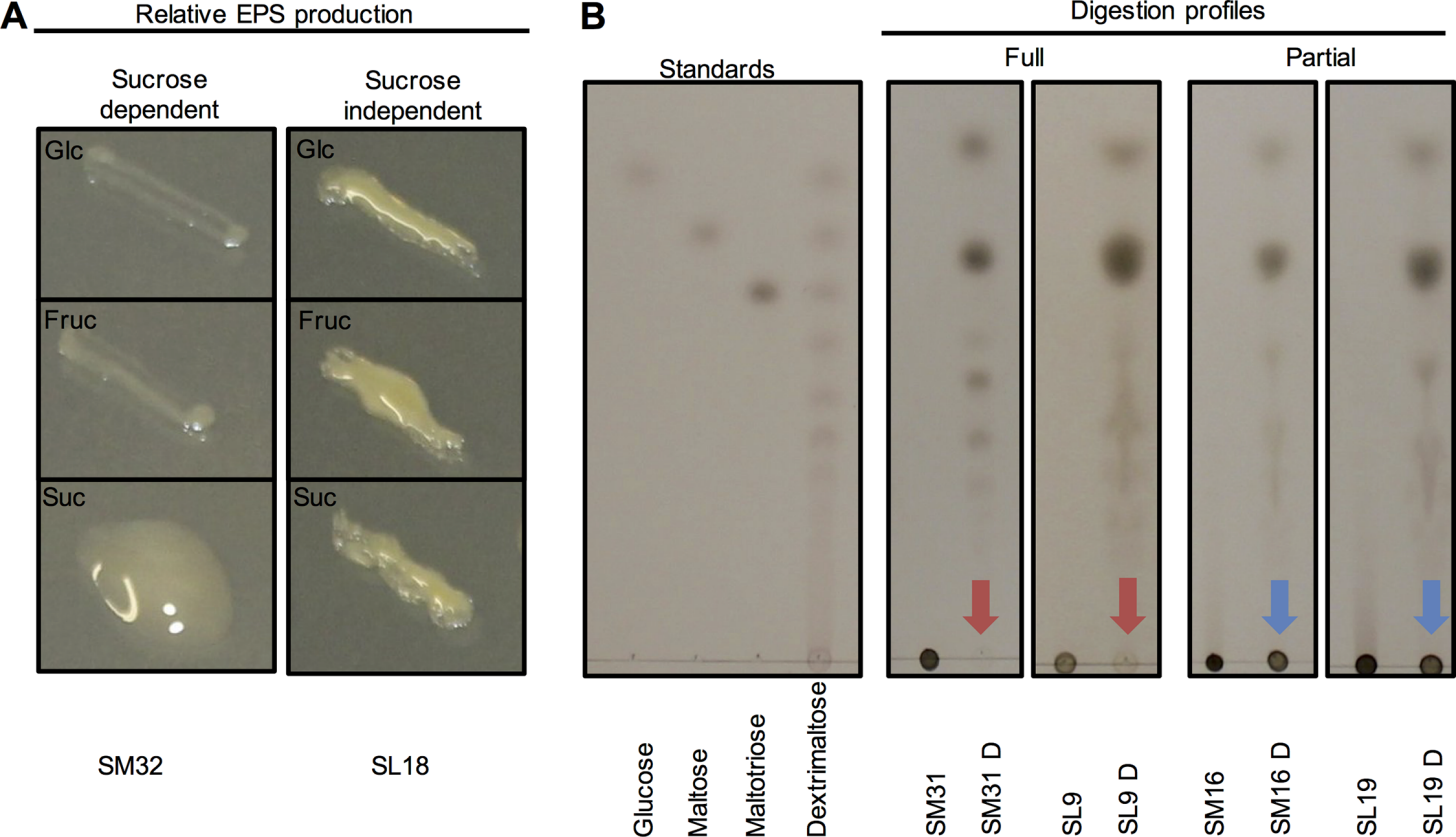
Polysaccharide accumulation and susceptibility to dextranase treatment. A–Sugar dependent or independent EPS production by bacteria grown on glucose, fructose or sucrose. B—Dextranase treatment of EPS. Full digestion is indicted by motile oligosaccharides in comparison to the undigested control; Partial digestion is indicated by an immobile spot at the origin in addition to motile oligosaccharides; Undigested EPS is indicated by no motile oligosaccharides.

Strict, sucrose dependent EPS formation (i.e. polysaccharide formation not observed when supplemented with glucose or fructose) was seen for the majority of isolates (Table 1, S1 Fig). Investigation of the composition of EPS, using GC-MS analysis of isolated and hydrolysed polymers, revealed that all 25 isolates produced glucose-containing polysaccharides. Mannose and fructose were also found in EPS produced by different *Leuconostoc* isolates, highlighting the diversity of EPS produced by a single species of bacteria. Strict, sucrose dependent polysaccharide formation is indicative of sucrase-type enzymes (i.e. dextran-, levan-, and inulosucrases) secretion (Fig 2A). These extracellular enzymes take advantage of the high energy sucrose glycosidic bond to produce glucose or fructose polymers, which offer environmental protection to the bacteria while releasing monomer sugars for uptake [33]. Interestingly, EPS from SL19 is comprised of glucose and fructose, possibly indicating the presence of both glucans and fructans produced by dextran- and fructansucrases, respectively. By contrast, the *S*. *bongori* like isolates produced EPS on all carbon sources tested (Fig 2A). These EPSs are, therefore, not likely produced via a sucrase enzyme but may involve the import of the sugar which is metabolised into an EPS exudate (Table 1, Fig 2). Touching a bacterial colony with a pipette tip or tooth-pick may produce a sticky string of EPS. This is known as the string test, a simple method of identifying EPS [33]. However, only *S*. *bongori* was string-test positive, easily capable of producing strings in excess of a meter (a string-test pass is 5mm), a property that may be attractive for biotechnological application [34]. This study revealed a diversity of polysaccharides with a range of physicochemical properties that are likely to interfere with downstream processing of sucrose.

A common strategy of EPS mitigation in mills, is the use of commercially available dextranase enzymes added to sugar “juice” after crushing. In order to test the efficacy of this approach, EPS from the 25 bacterial isolates were subjected to *in vitro* digestion by *Chaetomium erraticum* dextranase, a commercially available α-(1,6) glucosidic hydrolase. Susceptibility to dextranase digestion was evaluated and visualized by TLC (Fig 2B, S1 Fig). At least partial hydrolysis was demonstrated for all EPS, while only 12 showed complete digestion profiles. Complete digestion is scored when the TLC loading spot is nearly free from polysaccharide. The level of susceptibility to dextranase is directly correlated to the amount of enzyme-accessible linear α-(1,6) glucose units contained within the polysaccharides [14]. Dextran, an often poly-branched polyglucan, has been shown to have a predominance of α-(1,6) glycosidic linkages, in addition to having varying ratios of α-(1,2), α-(1,3) and α-(1,4) linkages [2, 15, 16]. This variation may contribute to the partial dextranase susceptibility seen in many of these isolates. Not surprisingly, heteropolysaccharides produced by SL19 were less amenable to enzymatic hydrolysis due to the presence of mannose or fructose. The presence of heteropolysaccharides will limit the effectiveness of dextranase enzymes. While dextranase treatment, a common remediating approach during milling, may reduce viscosity of the massecuite to some extent, the resulting release of oligosaccharides are known to negatively influence crystallisation of sucrose [8].

Dextransucrase is a glucosyltransferase (E. C. 2.4.1.5) that catalyses the transfer of glucosyl residues from sucrose to the dextran polymer and liberates fructose [15]. This enzyme is usually associated with *Leuconostoc*, *Weissella* and *Lactobacillus* spp, and is considered the primary causal agents of fouling in sugar mills [4, 35]. This study confirms the widespread consensus that dextran is the main polysaccharide derived from sugar deterioration by bacteria [4, 7, 34, 36, 37]. We, however, propose that the presence of polysaccharides, other than specifically dextran, may lead to significant processing complications and should be considered for their effect on milling, evaporation, and crystallisation of sugar.

## Conclusion

Numerous EPS-producing bacteria were isolated from sugarcane bagasse. This study revealed the presence of two genera novel to this niche with the ability to degrade sucrose. Polysaccharides isolated from the various sugarcane-associated bacteria revealed a complexity that has not been previously reported. Dextranase digestion was only partially effective against polysaccharide accumulation. The most effective approach in reducing the negative impact of bacterial polysaccharides remains sound management practices such as reducing the cut-to crush delay and proper mill sanitation.

## Supporting Information

S1 Fig EPS production of the various isolates when grown on glucose, fructose or sucrose supplemented media (upper panels). The EPS was purified and subjected to digestion by dextranase enzyme, the product of which was separated by Thin Layer Chromatography. These results are summarised in Table 1.
(TIFF)
