# Supplementary figures and images for: Diverse Exopolysaccharide Producing Bacteria Isolated from Milled Sugarcane: Implications for Cane Spoilage and Sucrose Yield
Source: PLoS One. 2015 Dec 28;10(12):e0145487. doi: 10.1371/journal.pone.0145487 (PMC4692551; doi:10.1371/journal.pone.0145487)

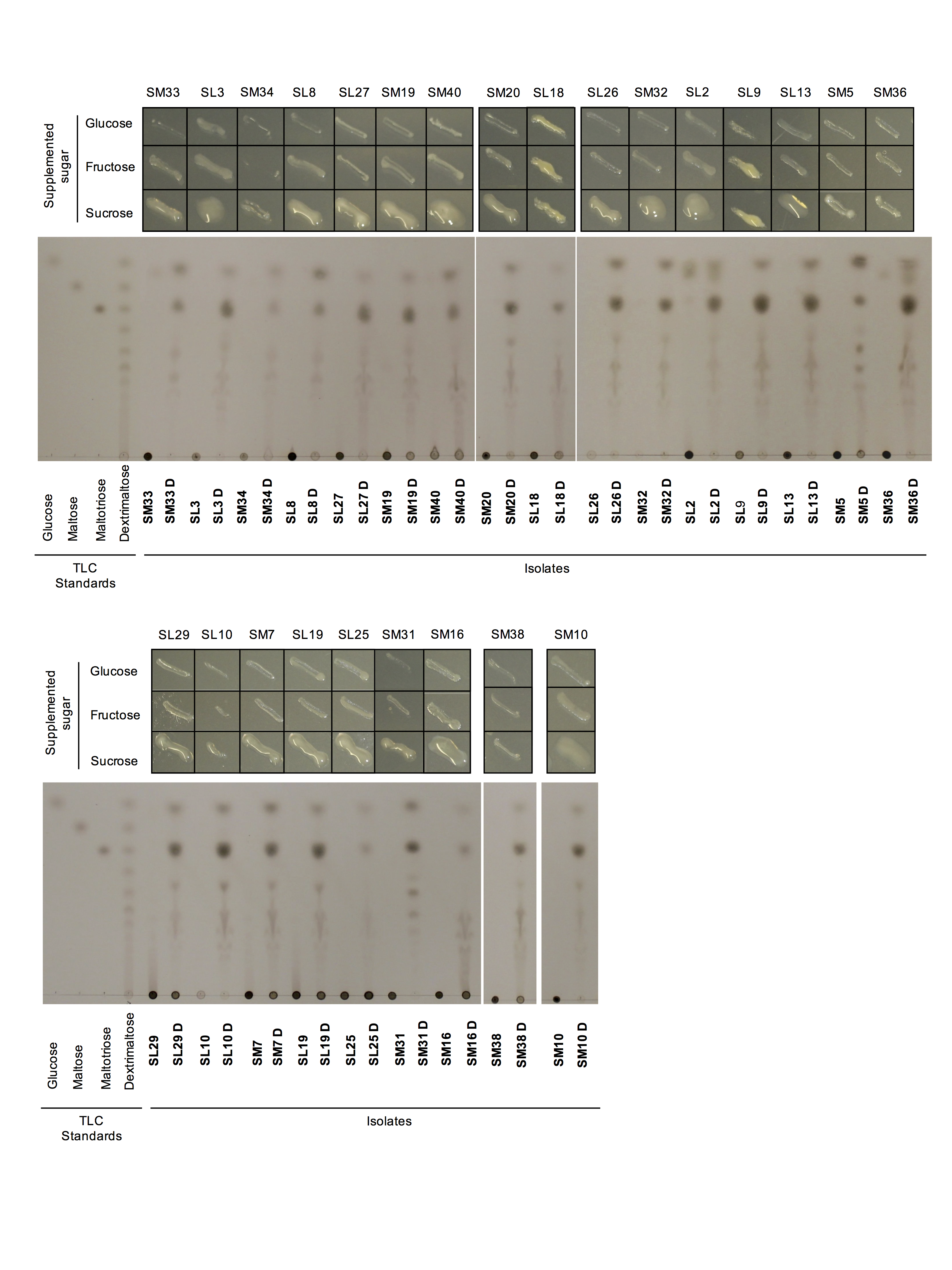

Supplement: S1 Fig — The EPS was purified and subjected to digestion by dextranase enzyme, the product of which was separated by Thin Layer Chromatography. These results are summarised in Table 1. (TIFF) [file pone.0145487.s001.tiff]
